# Development of a panel of DNA Aptamers with High Affinity for Pancreatic Ductal Adenocarcinoma

**DOI:** 10.1038/srep16788

**Published:** 2015-11-25

**Authors:** Carole Champanhac, I-Ting Teng, Sena Cansiz, Liqin Zhang, Xiaoqiu Wu, Zilong Zhoa, Ting Fu, Weihong Tan

**Affiliations:** 1 Department of Chemistry, Department of Biochemistry and Molecular Biology, Center for Research at the Bio/Nano Interface, Health Cancer Center, UF Genetics Institute and McKnight Brain Institute, University of Florida, Gainesville, Florida, USA; 2 Molecular Science and Biomedicine Laboratory, State Key Laboratory for Chemo/Bio Sensing and Chemometrics, College of Biology, College of Chemistry and Chemical Engineering and Collaborative Research Center of Molecular Engineering for Theranostics, Hunan University, Changsha, China

## Abstract

Pancreatic cancer costs nearly 40,000 lives in the U.S. each year and has one of the lowest survival rates among cancers. Effective treatment of pancreatic ductal adenocarcinoma is hindered by lack of a reliable biomarker. To address this challenge, aptamers were selected by cell-SELEX (Systematic Evolution of Ligands by EXponential enrichment) targeting human pancreatic ductal adenocarcinoma (PL45). Five promising aptamers presenting low K_d_ values and good specificity were generated. Among these five aptamers, one was tailored into a nanostructure carrying a high drug payload for specific drug delivery. The results show a viability of almost 80% for negative cells while only 50% of the target cells remained alive after 48 h incubation. These results lead to the conclusion that further research could reveal protein biomarkers specific to pancreatic adenocarcinoma, with probes available for early detection.

The pancreas is a very unique and complex organ composed of endocrine cells, responsible for the synthesis, storage and timely secretion of hormones (e.g., glucagon and insulin), and exocrine cells that produce pancreatic juice containing essential digestive enzymes^1^. Pancreatic cancer affects both cell types; however over 90% of pancreatic cancer can be attributed to exocrine tumour cells with 95% of these cases presenting adenocarcinoma *in situ*. Pancreatic cancer is the fourth leading cause of cancer- related death in the U.S. It has a very low 5 year survival rate (under 5%), with over 80% mortality within a year of diagnosis^2^,^3^,^4^,^5^. In 2014, this recalcitrant cancer cost nearly 40,000 lives in the U.S. and over 270,000 lives worldwide^2^. Lack of reliable biomarkers and specific symptoms, such as jaundice, loss of appetite and weight loss, at the early stage explain the poor prognosis. It takes several years for the disease to progress from the tumour initiating cells, PanIN (Pancreatic Intraepithelial Neoplasia) to metastasis, which, with early detection, would allow time for effective treatment^6^. The cell line chosen for this study, PL45, presents a point mutation of the KRAS gene at codon 12 along with a p53 gene mutation^7^. KRAS gene is frequently mutated in PanIN lesions, which are precursors of tumour cells, and the activation of KRAS leads to enhanced proliferation and cell growth. In addition, the inactivation of p53 gene is favoured by tumour cells, as this gene mediates cell cycle arrest. Patients presenting p53 and KRAS mutation typically have a low survival time^8^. Both cancer antigen 19-9 (CA 19-9) and carcinoembryonic antigen (CEA) are biomarkers of choice, but they are known to be unreliable before tumours reach the advanced metastatic stage. Thus, they are mainly used to follow treatment response^3^,^5^. Since, prognosis relies heavily on invasive and expensive techniques^4^,^5^, the discovery of a probe able to recognize pancreatic ductal adenocarcinoma at its earlier stage would represent a biomedical breakthrough. Aptamers are perfect candidates for this task based on their proven ability to distinguish specific markers on the cell surface^9^,^10^.

Aptamers are single-stranded oligonucleotides (DNA or RNA), that are capable of strong and specific binding to a target marker based on their unique three-dimensional folding^11^. Aptamers are often compared to antibodies since both demonstrate similar recognition mechanisms, specificity and selectivity. Aptamers are selected from an initially large oligonucleotide pool, containing 10^12^–10^15^ sequences, by a process called Systematic Evolution of Ligands by EXponential enrichment (SELEX)^12^,^13^. The SELEX process consists in an iterative enrichment of a pool of oligonucleotides towards a target. The pool and targets are incubated together to allow recognition of the target by some sequences. Then, the bound sequences are separated from the unbound sequences and finally, the bound sequences are amplified. Aptamers recognize a broad range of targets, including organic dyes^12^, small molecules^14^,^15^ and more recently cells^16^,^17^,^18^,^19^,^20^ and bacteria^21^. In this work, the target is a pancreatic ductal adenocarcinoma cell line (PL45). A negative cell line (TOV-21G) is introduced to remove sequences which bind to markers not specific to pancreatic adenocarcinoma, such as, for example, EpCAM protein, which is present in several types of cancer cells. Cell-SELEX technology targets whole living cells, expressing fully functional transmembrane proteins. Therefore transmembrane proteins of interest could be targeted by carefully choosing the cell line used^22^. Since no knowledge of the cell surface marker is required to obtain a cell-specific aptamer, new biomarkers can be readily discovered, presenting new horizons for potential *in vivo* applications.

In this work, eight DNA aptamers that recognize pancreatic ductal adenocarcinoma were selected and their binding characteristics, including specificity, affinity and internalization, were investigated. In addition, PL8 aptamer was engineered into a nanotrain nanostructure to carry and deliver doxorubicin specifically to pancreatic adenocarcinoma.

## Results and Discussion

### Generation of an enriched pool by cell-SELEX

The PL45 cell line was chosen as the model for pancreatic adenocarcinoma. The addition of a negative cell line (ovarian clear cell carcinoma, TOV-21G) provided a basis for removing sequences binding to common markers, such as EpCAM, expressed in most cancer cell lines. The selection started with the synthesis, by standard phosphoramidite chemistry^23^, of a library containing two 19 nucleotide (nt) primers (5′-FITC-CCAGCCTCAGACTCGGTGA-3′ and 5′-biotin-CGCTCGGATGCCACTACAG-3′) and a 25 nt random central region. In the first round, the initial library was incubated only with positive cells to remove the sequences having no affinity for the target cell. For subsequent rounds, the following four steps were repeated until enrichment was reached^24^. First, the pool obtained at the end of the previous round was incubated with the negative cells for one hour, and unbound sequences, those having no affinity for the negative cells, present in the supernatant were recovered. Second, this supernatant was incubated with the target cells. Third, the target cells were washed several times to remove loosely bound sequences, and the bound sequences were recovered by heat-induced dissociation. In the fourth step, surviving sequences were amplified by polymerase chain reaction (PCR), followed by purification. In the final step, the pool was sequenced. It is important to note that the first step was performed only for rounds 2–5, 7, 9, 11, 13, 15 and 18–22.

**Figure 1:**
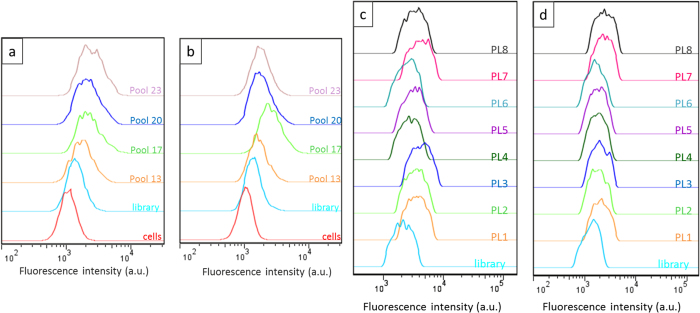
Flow cytometry showing progressive enrichment in binding sequences for the positive cell line (a), while the enrichment towards the negative cell line (b) was reduced by round 17. Binding affinity of the aptamers (250 nM) for PL45 cells at 4 °C (**c**) and 37 °C (**d**).

Enrichment was monitored by flow cytometry. The enrichment of the pool is shown by an increase in fluorescence intensity indicating an increase in bound FITC-labelled DNA sequences on the cell surface. As shown in Fig. 1a, the library shows enrichment for the positive PL45 cell line between rounds 13 and 20, reaching a plateau throughout round 23. The pools generated from rounds 13 to 17 showed some affinity for the negative cell line (TOV-21G) (Fig. 1b); however, an increase in selection stringency by increasing the negative cell concentration and implementing harsher washing conditions was able to reduce this nonspecific binding to a minimum at round 20. Pool 23 was considered the best candidate for sequencing and was sent for Ion Torrent^TM^ Next-Generation sequencing.

### Binding affinity of the panel of aptamer candidates

The raw data received from the sequencing process provided two million sequences. The analysis of these sequences was performed by software made in house, termed DNASeAl, and revealed the presence of several identical sequences. These sequences were grouped and the eight most abundant were synthesized for further characterization. These sequences, along with their relative abundance and equilibrium dissociation constant (K_d_), are listed in Table 1. Binding assays were conducted on cells at various passaging and no significant variation in the binding profile was observed. Aptamers PL1, PL2, PL3, PL7 and PL8 strongly bound PL45 cells both at 4 °C and at physiological temperature. On the other hand, aptamer PL4 exhibited a weak binding and PL6 showed a very weak binding at both temperatures, thus limiting their potential for future applications. Finally aptamer PL5 showed strong binding at 4 °C but weaker binding at 37 °C (Fig. 1c,d). The weaker fluorescence signal could be explained by a lower abundance of target on the cell surface or by unstable folding of the aptamer, although the latter explanation is inconsistent with the K_d_ values obtained for PL4 and PL5. As expected, the selected aptamer showed no toxicity to cells, making this probe ideal for recurrent detection (Supplementary Fig. S1).

Equilibrium dissociation constants (K_d_) were obtained by incubating the target cells with aptamer molarities ranging from 0.12 nM to 1000 nM. Overall the equilibrium dissociation constants are under 50 nM for most aptamers (Table 1). Temperature seems to have had little impact on most of them. It is interesting to note that PL2 had a better K_d_ at 4 °C while PL8 had a better K_d_ at 37 °C. PL6 exhibited surprising loss of its binding ability at 37 °C as can be seen by its high K_d_, most likely resulting from the loss of its tertiary structure. Indeed, a lower K_d_ translates into tighter binding due to a better folding of the aptamer around its target, underlining the importance of forming a stable tertiary structure.

### Specificity of the selected aptamer

The aptamers were tested against nine cancer cell lines and one normal cell line to determine their specificity (Table 2). No affinity was demonstrated for lung carcinoma (A549 and H226), prostate carcinoma (DU 145), lymphoma (Ramos) or leukaemia (CCRF-CEM) at 37 °C. The same binding profile was obtained for PL45 and Hep G2 cells implying the presence of at least one common marker. However, most aptamers, except for PL7, showed little or no affinity for normal pancreatic cells (hTERT-HPNE) at 37 °C. This is a major advantage for future *in vivo* applications like detection or drug delivery. Aptamer specificity was also investigated at 4 °C, and a similar trend in terms of binding affinity was observed (Supplementary Table S1).

### Cellular uptake of aptamers

**Figure 2:**
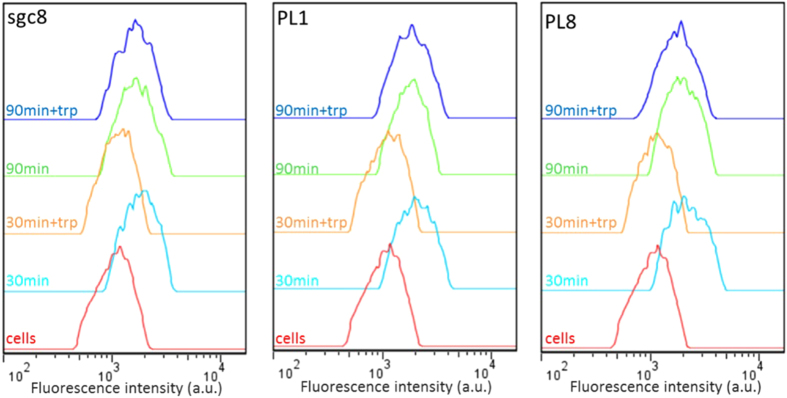
Internalization of PL1 and PL8. Sgc8 is used as a positive control. The cells are incubated at first for 30 min at 4 °C, after dye staining, the incubation is continued for 90 min at 37 °C. The cells are then treated with trypsin (30 min + trp or 90 min + trp).

A key feature of the generated aptamers is their internalization ability. As shown in Fig. 2, PL1 and PL8 presented a right shift in fluorescence intensity after 30 minutes incubation at 4 °C. If the incubation is continued for 90 minutes at 37 °C, the shift is maintained. However, upon trypsin treatment, the signal is lost for the cells incubated for 30 minutes but retained for those incubated for an extra 90 minutes. The loss of the signal after 30 min incubation followed by trypsin treatment demonstrates that the target of the aptamer is a protein. Meanwhile, retention of the signal after 90 minutes of incubation and trypsin treatment indicates that aptamers have been internalized by the cells. The same behaviour is observed for aptamers PL2, PL3 and PL7, along with aptamer sgc8, which is known to be internalized^25^, while a random library presents a back shift after 30 minutes and 90 minutes of incubation followed by trypsin treatment (Supplementary Fig. S2). The natural uptake of these aptamers is a major advantage since aptamer-drug conjugates depend on cell internalization for efficacy.

### Engineering a high-affinity aptamer into a nanostructure for drug delivery

**Figure 3:**
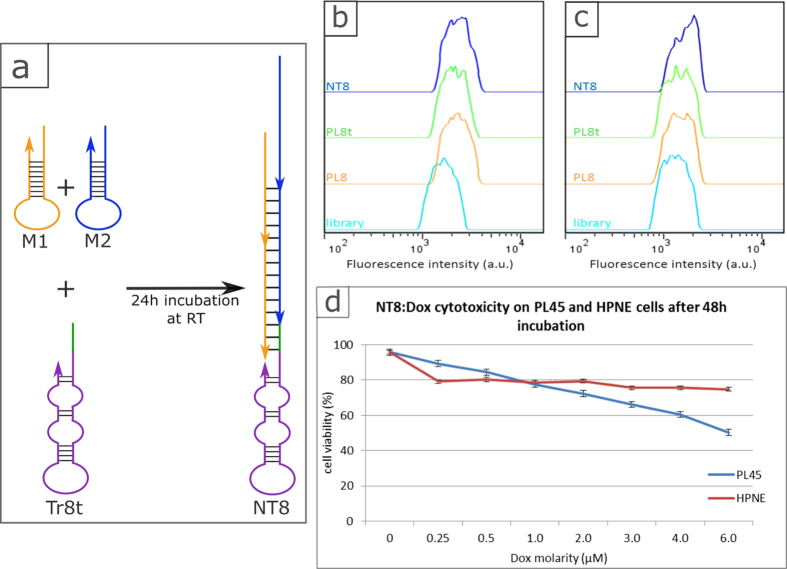
Binding of PL8t (shorter version of PL8) and NT8 at 37 °C. (**a**) Schematic of nanotrain formation: aptamer PL8t is represented in purple. The trigger is in green and the monomers M1 and M2 are respectively in orange and blue. (**b**) PL8, PL8t and NT8 show similar binding affinity towards PL45 cells. (**c**) PL8 and PL8t show no affinity for hTERT HPNE; however NT8 shows some affinity for these cells. (**d**) PL45 (target) and HPNE (control) cells were incubated for 2 h with the doxorubicin loaded nanotrain (NT8:Dox) and cultured thereafter for 48 h. A dose dependent response was achieved for the target cells, while the control cells showed a constant viability (above 75%). The viability at 0 μM represents the viability of cells incubated with only NT8.

Since aptamers are chemically synthesized by successive addition of each base, it is accepted that the shortest oligonucleotide will give the best overall yield. Therefore, aptamer PL8, presenting a low K_d_ at 37 °C (26 nM) and a strong binding to pancreatic adenocarcinoma but no affinity for normal pancreatic cells, was chosen to be truncated into a 45 nt long aptamer, termed PL8t. In comparison with parent PL8, PL8t displays similar binding ability for cell line of interest, PL45 (Fig. 3b), and normal cells, hTERT HPNE (Fig. 3c).

Then, inspired by work previously performed in our laboratory^26^, aptamer PL8t was chosen as the recognition moiety for the construction of a nanotrain, which is a three-dimensional structure composed of partially complementary short DNA strands (M1 and M2) hybridized together to form a long chain of “box cars” to transport a large payload, such as an imaging agent or drug. If M1 and M2 are mixed together no reaction occurs; however, the addition of the aptamer modified with a trigger sequence (Tr8t) induces the formation of a nanotrain (NT8) (Fig. 3a and Supplementary Fig. 3a). M1 and M2 are designed so that Doxorubicin (Dox), a chemotherapeutic drug, can be intercalated and carried to the target cells (Supplementary Fig. 3b). The sequences of M1, M2, Tr8t and PL8t are provided in Supplementary Table S2. Retention of the binding affinity was investigated, and compared to the binding profile of PL8, NT8 showed comparable binding affinity, although it displayed a larger shift compared to PL8 with hTERT HPNE cells. This behaviour could be expected, as nonspecific interactions are stronger for the nanostructure (Fig. 3b,c). In addition, NT8 was demonstrated to be internalized by pancreatic adenocarcinoma cells (Supplementary Fig. S3c), a requisite for drug delivery.

In order to determine the efficacy of NT8 for drug delivery, both target cells (PL45) and control cells (hTERT HPNE) were treated with free Dox (positive control) and drug-loaded nanotrain (NT8:Dox). The viability of the cells was determined after 48 h incubation by MTS assay (Fig. 3d and Supplementary Fig. S4a,b). As demonstrated in a previous study^26^, the drug is released from the nanotrain by diffusion assisted by the change of pH in the cellular compartments and the presence of nucleases degrading the nanostructure. NT8:Dox showed dose-dependent cytotoxicity for the target cells, while the viability of the control cells remained constant and above 75%, despite an increase in drug concentration. At low doxorubicin molarity, NT8:Dox was less efficient in killing adenocarcinoma compared to free Dox. However, as the molarity increased, the gap between NT8:Dox and free Dox decreased and disappeared at 6 μM (Supplementary Fig. S4a). On the other hand, NT8:Dox displayed reduced toxicity to normal pancreatic cells even at high dosage (Supplementary Fig. S4b), it is an important criterion to control side effects. The limited toxicity induced by the drug-loaded nanotrain in contact with normal cells is a very promising result, as it proves the retention of aptamer specificity even after modification into a nanostructure.

## Conclusion

We have successfully generated eight aptamers with high affinity for pancreatic ductal adenocarcinoma. Five of these aptamers exhibit strong binding toward the PL45 cells, while three have a weaker binding affinity. Overall, the aptamers are specific to pancreatic adenocarcinoma, despite some affinity towards hepatocellular carcinoma. The main features of these aptamers are low equilibrium dissociation constants (30–50 nM), which demonstrate tight binding of the aptamer to the cell membrane target protein and internalization by the target cancer cells. In addition, aptamer PL8 was successfully engineered to serve as the recognition moiety for a three-dimensional nanotrain structure carrying a high-capacity payload of anticancer drug. Low cytotoxicity to healthy cells indicates that *in vitro* results should be further tested *in vivo* to assess drug release kinetics and accumulation sites. Even though the identity of markers on the surface of these cells remains unknown at this time, our results suggest that further research could reveal protein biomarkers specific to pancreatic adenocarcinoma enabling the development of probes for the early detection of pancreatic cancer.

## Methods

Unless otherwise specified every reagent was purchased from Sigma-Aldrich. In addition, all flow cytometry and viability tests were performed in duplicate.

### Buffers

Washing buffer (WB) was prepared by dissolving glucose (4.5 g/L) and magnesium chloride (5 mM) in Dulbecco’s phosphate buffered saline. Binding buffer (BB) was prepared by adding bovine serum albumin (1 mg/mL) and Transfer ribonucleotide acid (tRNA) (0.1 mg/mL) to washing buffer in order to limit nonspecific binding. Polymerase chain reaction (PCR) reagents were purchased from TaKaRa. A table summarizing the amount of each reagent used and the protocol is presented in the Supplementary Table S3.

### Cell culture

PL45 (pancreatic ductal adenocarcinoma (CTRL-2558)), HeLa (cervix adenocarcinoma (CCL-2)), DU 145 (prostate carcinoma (HTB-81)) and Hep G2 (hepatocellular carcinoma (HB-8065)) were purchased from American Type Culture Collection (ATCC) and maintained in Dulbecco’s Modified Eagle’s Medium (DMEM) supplemented with sodium bicarbonate (1.5 g/L). TOV-21G (ovarian clear cell carcinoma) was purchased from ATCC (CTRL-11730) and maintained in a 1:1 mixture of MCDB 105 medium (1.5 g/L sodium bicarbonate) and Medium 199 (2.2 g/L sodium bicarbonate). Ramos (Burkitt’s lymphoma (CTRL-1596)), CCRF-CEM (acute lymphoblastic leukaemia (CRM-CCL-119)), A549 (lung carcinoma (CCL-185)) and H226 (lung squamous cell carcinoma (CRL-5826)) were purchased from ATCC and maintained in RPMI-1640 medium. hTERT HPNE (normal pancreatic ductal cell (CRL-4023)) was purchased from ATCC and maintained in recommended media (1:3 mixture M3:Base F^TM^ Culture Media: DMEM). All media were supplemented with heat inactivated fetal bovine serum (10% v/v (Gibco)) and penicillin-streptomycin (100 UI/mL (Gibco)). All cells were cultured at 37 °C in a 5% CO_2_ atmosphere. In addition to maintain the integrity of the cell lines and prevent mutation of the cells, the cells passaging were kept under 75.

### DNA synthesis and purification

An ABI 3400 DNA synthesizer (Applied Biosystems) was used to synthesize the initial single-stranded DNA library (5′-CCA GCC TCA GAC TCG GTG A (N)_25_C TGT AGT GGC ATC CGA GCG-3′ ) along with the selected aptamers. The products were purified by reversed phase HPLC (ProStar, Varian) using a C18 column and a linear elution gradient of acetonitrile:trietylammonium acetate. After deprotection of the trityl group, HPLC-purified products were dried and suspended in water. The concentration was determined by UV-vis spectrophotometry (Beckman Coulter DU800) at 260 nm. The primers used were ordered from Integrated DNA Technology.

### Cell-SELEX

For the first round, 13 nmol of the single-stranded library was incubated with PL45 cells for one hour at 4 °C. In subsequent rounds, only 200 pmol of the previous pool was incubated with the cells. The DNA solution was incubated first with the negative cells for one hour at 4 °C on an orbital shaker. The supernatant was recovered and incubated with the positive cells at 4 °C. They were washed several times with ice cold washing buffer. The cells were then scraped off the dish and eluted with binding buffer. This mixture was heated at 95 °C forcing the DNA to unfold. To separate the cell debris from the DNA sequences, the solution was spun down at 14000 rpm. The stringency of the selection was increased by shortening the incubation time of the positive cells from one hour to half an hour in the first four rounds; in addition the number of positive cells was decreased. The duration of the washing step was also increased from 1 to 3 minutes and the number of washes increased from two to six.

The DNA solution obtained after centrifugation was amplified by PCR (BioRad T100 or C1000 ThermoCycler). At first, the number of cycles was optimized and a 3% agarose gel stained with 0.06% v/v of ethidium bromide was used to verify the quality of the amplified product (revealed by UV exposure). The cycle displaying the brightest band without smear was chosen for amplification of the DNA solution. The recovery of the sense strand was done by passing the PCR solution through a column packed with streptavidin-coated Sepharose beads (GE Healthcare). The sense strand was labelled with fluorescein and the antisense strand bearing a biotin tag was retained. A basic solution of sodium hydroxide was passed through the column to cut the hydrogen bonds between the strands and permit elution of the sense strand. This solution was then passed through a NAP5 column for desalting, concentrated to 1.0 μM and stored at −20 ˚C. A complete description of the process can be found elsewhere^24^.

### Enrichment

To follow the evolution of the selection process, the binding ability of each pool was tested by flow cytometry. In brief, cells were detached from the dish with a non-enzymatic cell dissociation buffer and their viability was verified by trypan blue staining. A total of 300,000 living cells were incubated for 30 minutes at 4 °C with 25 pmol of each ssDNA pool generated. After incubation, the cells were washed twice with WB and suspended in BB for fluorescence detection by the Accuri C6 flow cytometer (BD Biosciences). Enrichment was proven by a right shift of fluorescence intensity. Both positive (PL45) and negative (TOV-21G) cells were tested to evaluate the specificity of the pool.

### Sequencing

Ion Torrent^TM^ Next-Generation DNA sequencing was used to determine the sequences present in the enriched pool. In brief, the selected pool was first amplified with specific fusion primers (trP1-forward and A-reverse), as recommended by Life Technologies. After purification, the sample was submitted to sequencing performed by the ICBR Core Facility, Genomics Division, University of Florida.

### Flow cytometry

The binding affinity of the aptamer candidates was determined by incubating 300,000 PL45 cells with a 50 pmol solution of biotinylated aptamer in binding buffer for 30 minutes at 4 °C or 37 °C. The cells were then washed twice with ice cold WB and further incubated with a diluted solution of streptavidin-PE-Cy5.5 dye (Life Technologies) for 15 minutes at 4 °C. They were washed again twice with WB and finally suspended in BB for fluorescence detection. A total of 40,000 events were recorded per sample and the data were analysed by FlowJo software.

In order to determine the equilibrium dissociation constants (K_d_), the cells were incubated with fourteen different aptamer molarities ranging from 1000 nM to 0.12 nM. The binding test was performed as described above. The mean fluorescence intensity of a random library was subtracted from the measured intensity for each molarity of the aptamer in order to account for nonspecific binding. This difference in fluorescence intensity was plotted against the molarity and a nonlinear regression (Y = B*x/(K_d_+x)) was fitted on the graph with SigmaPlot software, where Y represents the mean fluorescence intensity, B represents the saturated binding fluorescence intensity, x represents the molarity (mol/L) and K_d_ represents the dissociation constant (mol/L).

The internalization procedure was as follows: A binding affinity test was performed by incubating the cells at 4 °C for 30 minutes with PE-Cy5.5-labelled aptamers. From that point on, the cells were further incubated for 1h30 at 37 °C in BB then washed twice with WB. The trypsin treatment consisted of incubating the cells with 300 μL trypsin at room temperature for 20 minutes, followed by the addition of FBS to inhibit trypsin activity. Finally, the cells were washed twice with WB.

### Aptamer cytotoxicity

Cytotoxicity was determined using the CellTiter 96^®^ Aqueous One Solution Cell Proliferation Assay (Promega), following the procedure recommended by the manufacturer^27^. Solutions of 5.0 μM, 2.0 μM, 1.0 μM or 0.5 μM of the aptamer or doxorubicin were incubated with the cells in a FBS-free medium for 2 hours at 37 °C. The cells were further cultured for 48 h in complete media at 37 °C in 5% CO_2_. After removing the medium, MTS reagent (20 μL) diluted in 100 μL media was added and incubated for 2 to 4 hours. The absorbance (490 nm) was recorded using a plate reader and the viability was determined as described by the manufacturer.

### Nanotrain construction (NT8)

The nanotrain is composed of 3 parts: two short DNA sequences (M1 and M2) used to elongate the nanotrain and a probe part (Tr8t) used to recognize the target cells and trigger the formation of the nanotrain. In brief, Tr8t, M1 and M2 were thawed at 4 °C and then denatured for 3 min at 95 °C, snapped cool for 3 min at 4 °C and finally allowed to adjust to room temperature for 2 h. Tr8t, E1 and E2 were mixed and left to react for 24 h at room temperature, resulting in the formation of a nanotrain structure. A detailed procedure can be found elsewhere^26^,^28^.

### Dox-loaded nanotrain (NT8:Dox)

Upon the formation of the nanotrain structure, doxorubicin (Dox) (50:1 molar ratio) was added to the mixture and allowed to react for 1 h at room temperature. The solution was dialyzed for 14 h at 4 °C in PBS buffer containing 5 mM MgCl_2_ to remove any excess Dox. *In vitro* cytotoxicity was determined by CellTiter 96^®^ Aqueous One Solution Cell Proliferation Assay (Promega).A total of 5000 cells were treated with free Dox, Dox-loaded on NT8 (NT8:Dox) and NT8 in FBS free media for 2 hours at 37 °C. Then, 80% of the solution was removed and replaced with complete media and further cultured for 48 h. After removing the medium, MTS reagent (20 μL) diluted in 100 μL media was added to the cells and incubated for 2 h to 4 h. The absorbance (490 nm) was recorded using a plate reader and the viability was determined as described by the manufacturer.

## Additional Information

**How to cite this article**: Champanhac, C. *et al.* Development of a panel of DNA Aptamers with High Affinity for Pancreatic Ductal Adenocarcinoma. *Sci. Rep.* **5**, 16788; doi: 10.1038/srep16788 (2015).

## Supplementary Material

Supplementary Information

## Figures and Tables

**Table 1:** Compendium of aptamers generated by selection against PL45 cells.

Aptamers	Sequence	Relative abundance	K_d_ ± s.d. (nM) at 4 °C	K_d_ ± s.d. (nM) at 37 °C
**PL1**	5′-CGC TCG GAT GCC ACT ACA GTG CTA ATC TCA AGG GTC GTT CCC GAT CAC CGA GTC TGA GGC TGG-3′	8.88%	28.4 ± 3.6	24.9 ± 3.6
**PL2**	5′-CGC TCG GAT GCC ACT ACA GGA ACT AAC ACA CTA CTG AAC CGT GCT CAC CGA GTC TGA GGC TGG-3′	8.24%	8.3 ± 2.5	20.9 ± 3.5
**PL3**	5′-CGC TCG GAT GCC ACT ACA GCA CTC ACC TCA AGG GTT CCG TGT CAC CGA GTC TGA GGC TGG-3′	7.55%	16.2 ± 3.0	23.1 ± 5.3
**PL4**	5′-CGC TCG GAT GCC ACT ACA GGG ACT AAG CAC ACT ACT GTT CAC GGT CAC CGA GTC TGA GGC TGG-3′	5.51%	16.6 ± 6.5	11.9 ± 1.4
**PL5**	5′-CGC TCG GAT GCC ACT ACA GCC AGC GTG GAT ATG GGT TCC ACT GGT CAC CGA GTC TGA GGC TGG-3′	2.75%	55.3 ± 4.4	65.5 ± 18.1
**PL6**	5′-CGC TCG GAT GCC ACT ACA GTA CAC ACT GGT CTC AAG GGT GTG AGT CAC CGA GTC TGA GGC TGG-3′	2.49%	47.3 ± 16.0	178.9 ± 30.1
**PL7**	5′-CGC TCG GAT GCC ACT GTT GAG GTG TAT TGT ACA CGT GGG GTT ACA CAC CGA GTC TGA GGC TGG-3′	3.00%	43.3 ± 13.8	30.0 ± 11.2
**PL8**	5′-CGC TCG GAT GCC ACT ACA GCA TAT ATC CTC CCC CCA TGC GTG GTC ACC GAG TCT GAG GCT GG-3′	2.09%	60.9 ± 15.5	26.1 ± 6.5

**Table 2:** Specificity of aptamers towards different cell lines tested at 37 °C.

	PL1	PL2	PL3	PL4	PL5	PL6	PL7	PL8
PL45	+++	++	++	++	++	−	+++	+++
TOV-21G	−	−	+	−	−	+	−	+
hTert HPNE	−	−	+	+	−	−	++	−
Hep G2	+++	+++	+++	+	−	+	++	+++
HeLa	+	++	+++	−	−	−	−	−
A549	−	−	−	−	−	−	−	−
H226	−	−	−	−	−	−	−	−
DU 145	−	−	−	−	−	−	−	−
CCRF-CEM	−	−	−	−	−	−	−	−
Ramos	−	−	−	−	−	−	−	−

A minus (−) sign means affinities of 0–10% for the cells, a plus (+) means affinities of 10–40%, (++) means affinity of 41–70% and (+++) means affinity of 71–100%.
