# Supplementary material for: Development of a panel of DNA Aptamers with High Affinity for Pancreatic Ductal Adenocarcinoma
Source: Sci Rep. 2015 Nov 25;5:16788. doi: 10.1038/srep16788 (PMC4658478; doi:10.1038/srep16788)
Supplement: Supplementary Information [file srep16788-s1.doc]

**Supplementary Information**

**Development of a panel of DNA Aptamers with High Affinity for Pancreatic Ductal Adenocarcinoma**

Champanhac, Carole^1^; Teng, I-Ting^1^; Cansiz, Sena^1^; Zhang, Liqin^1^; Wu, Xiaoqiu^2^; Zilong, Zhao^2^; Ting, Fu^2^ and Tan, Weihong^1,2*^

1. Department of Chemistry, Department of Biochemistry and Molecular Biology, Center for Research at the Bio/Nano Interface, Health Cancer Center, UF Genetics Institute and McKnight Brain Institute, University of Florida, Gainesville, Florida, USA
2. Molecular Science and Biomedicine Laboratory, State Key Laboratory for Chemo/Bio Sensing and Chemometrics, College of Biology, College of Chemistry and Chemical Engineering and Collaborative Research Center of Molecular Engineering for Theranostics, Hunan University, Changsha, China

* Corresponding author: [tan@chem.ufl.edu](mailto:tan@chem.ufl.edu)

**Supplementary Information:**

Figure S1: Determination of aptamers cytotoxicity by MTS assay

Figure S2: Internalization test for a random library (negative control), PL2, PL3 and PL7 aptamers

Figure S3: Nanotrain structure formation confirmation (a), Schematic of drug loading in the nanotrain (b), Verification of the internalization of NT8 by PL45 cells (c)

Figure S4: Cytotoxicity of doxorubicin-loaded nanotrains compared between control cells (a) and target cells (b)

Table S1: Specificity of the aptamers at 4˚C

Table S2: Nanotrain DNA sequences

Table S3: Polymerase chain reaction reagents and procedure details

**Supplementary figures:**


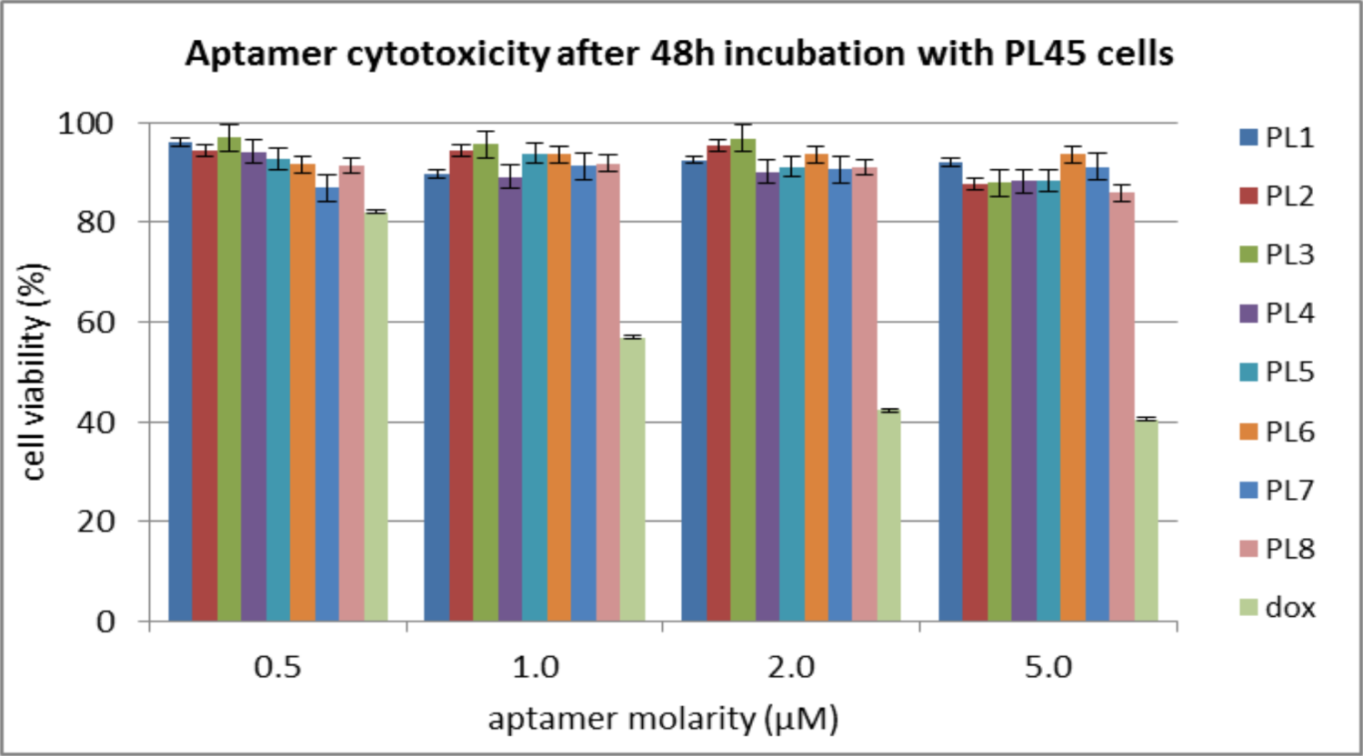


Supplementary Figure S1: Determination of PL45 cell viability by MTS assay after 4h incubation with the aptamer and further 48h culture in complete media. The assay is validated by the presence of free doxorubicin as a positive control to assess the nontoxicity of aptamers for the target cells.


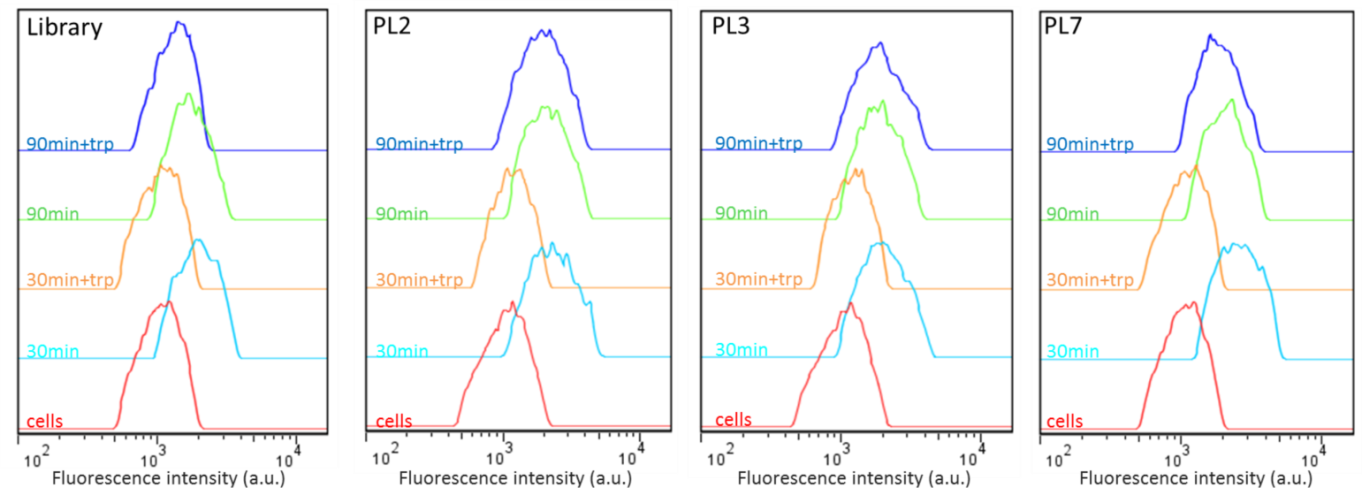


Supplementary Figure S2: Internalization test for a random library (negative control), PL2, PL3 and PL7 aptamers. The cells were incubated for 30 minutes at 4˚C and then treated with trypsin or incubated for 90 minutes at 37˚C followed by trypsin treatment. A back shift is clearly visible following trypsin treatment and 30 minutes incubation in all cases. However, only library presents a significant back shift after trypsin treatment and 90 minutes incubation at 37˚C. Therefore, the aptamers were internalized by cells after an incubation of 1h30min at 37˚C while random library was not internalized by the cells under the same conditions.


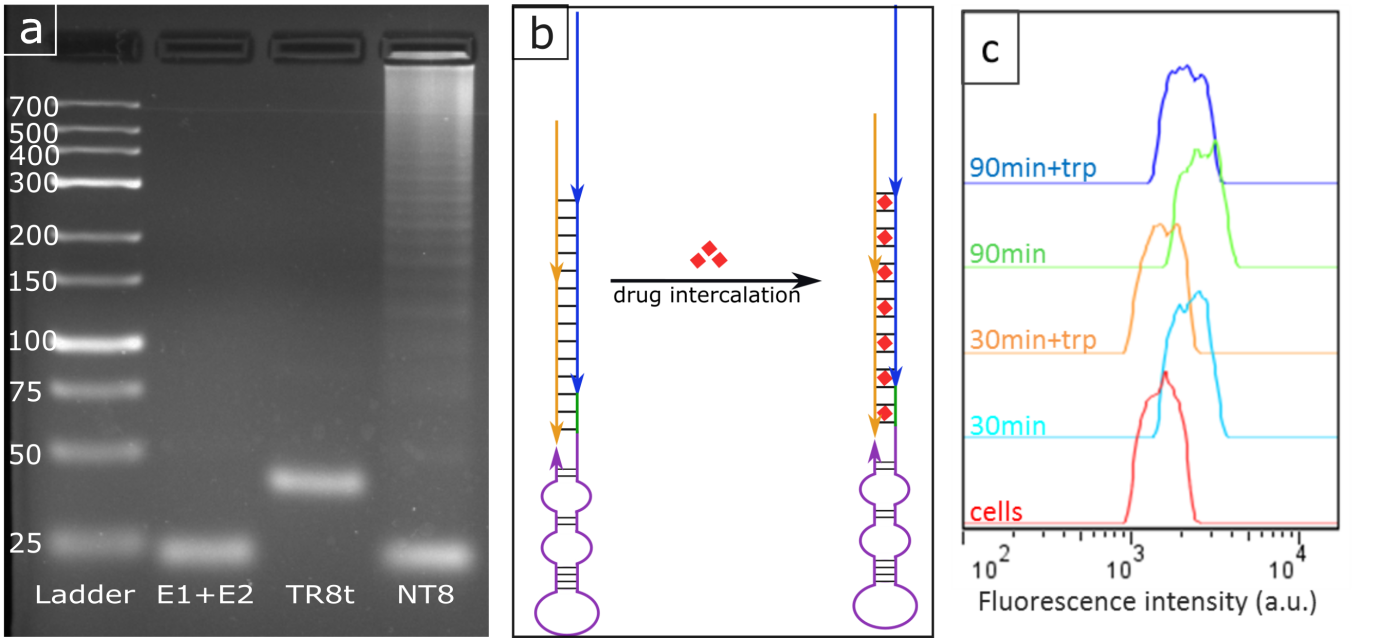


Supplementary Figure S3: (a) Verification of the formation of a nanostructure (NT8) by gel electrophoresis. The smear on top of the NT8 column proves the formation of a structure too big to move in a 3% agarose gel. (b) Schematic of nanotrain drug loading. (c) Verification of the internalization of NT8 by PL45 cells. Despite a slight backshift, most of the binding is retained for the cells incubated for 90min with trypsin treatment compared to cells incubated for 90 minutes indicating the internalization of the nanotrains.


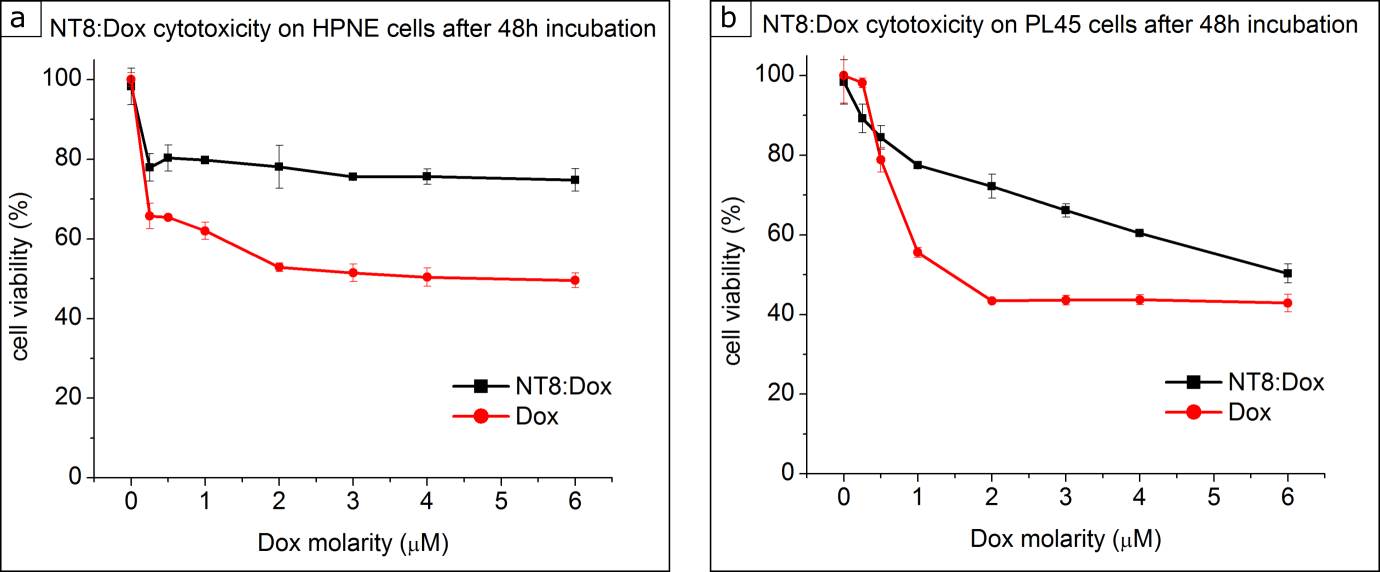


Supplementary Figure S4: Cytotoxicity of doxorubicin-loaded nanotrains compared between control cells (a) and target cells (b).For PL45 cells, the cytotoxicity of NT:Dox increases in a dose-dependent manner, reaching the same efficiency as free drug for a high dosage, while displaying reduced toxicity compared to free drug for control cells.

**Supplementary Tables:**

|  | PL1 | PL2 | PL3 | PL4 | PL5 | PL6 | PL7 | PL8 |
| --- | --- | --- | --- | --- | --- | --- | --- | --- |
| PL45 | ++++ | +++ | ++++ | + | ++ | + | ++++ | +++ |
| TOV-21G | + | ++ | ++ | - | - | - | - | + |
| HeLa | - | - | - | - | - | - | - | - |
| A549 | - | - | - | - | - | - | - | - |
| H226 | - | - | - | - | - | - | - | - |
| HepG2 | + | + | ++ | + | - | + | + | +++ |
| DU 145 | - | - | - | - | - | - | - | - |
| CEM | - | - | - | - | - | - | - | - |
| Ramos | - | - | - | - | - | - | - | - |

Supplementary Table S1: Specificity of the aptamers at 4˚C. A minus (-) sign indicates affinity from 0-10%, (+) indicates affinity from 11-35%, (++) indicates affinity from 36-60%, (+++) indicates affinity from 61-80% and (++++) indicates affinity from 81-100%.

| **Oligonucleotide** | **Sequence** |
| --- | --- |
| **PL8t** | 5’ - CGG ATG CCA CTA CAG CAT ATA TCC TCC CCC CAT GCG TGG TCA CCG -3’ |
| **Tr8t** | 5’-TGC TGC TGC TGC TGC TGC ACG ACG TTT CGG ATG CCA CTA CAG CAT ATA TCC TCC CCC CAT GCG TGG TCA CCG -3’ |
| **M1** | 5’- CGT CGT GCA GCA GCA GCA GCA GCA ACG GCT TGC TGC TGC TGC TGC TGC -3’ |
| **M2** | 5’- TGC TGC TGC TGC TGC TGC ACG ACG GCA GCA GCA GCA GCA GCA AGC CGT -3’ |

Supplementary Table S2: For Tr8t, purple indicates the aptamer and green indicates the trigger probe. Tr8t is labelled with biotin on the 3’ end.

| **Reagents** | **50μL** | **1000μL** |
| --- | --- | --- |
| 10x buffer | 5.00 | 100.0 |
| dNTP mixture (2.5mM each) | 4.00 | 80.0 |
| forward and reverse primers mix | 2.50 | 50.0 |
| Taq polymerase | 0.15 | 3.0 |
| template | 5.00 | 100.0 |
| water | 33.35 | 670.0 |

Supplementary Table S3: Polymerase chain reaction reagents used for the cycle optimization step (50μL) and the amplification step (1000μL). Each optimization cycle was performed at 95˚C for 30s, then 8-16 repeats of 30s at 95˚C, 30s at 64˚C and 30s at 72˚C, with a final extension of 5min at 72˚C.
